# Cigarette Smoking Among Pregnant Women During the Perinatal Period: Prevalence and Health Care Provider Inquiries — Pregnancy Risk Assessment Monitoring System, United States, 2021

**DOI:** 10.15585/mmwr.mm7317a2

**Published:** 2024-05-02

**Authors:** Lauren Kipling, Jennifer Bombard, Xu Wang, Shanna Cox

**Affiliations:** ^1^Division of Reproductive Health, National Center for Chronic Disease Prevention and Health Promotion, CDC; ^2^Office on Smoking and Health, National Center for Chronic Disease Prevention and Health Promotion, CDC.

**Summary**

**What is already known about this topic?**

Cigarette smoking has wide-ranging adverse health consequences, and when it occurs during pregnancy, there are increased risks of pregnancy complications and adverse outcomes for infants.

**What is added by this report?**

In 2021, among women with a recent live birth, 12.1% reported smoking before pregnancy, 5.4% reported smoking during pregnancy, and 7.2% reported smoking during the postpartum period. Smoking behaviors varied by demographic characteristics and jurisdiction. Overall, 73.7%, 93.7%, and 57.3% of women reported being asked about smoking by a health care provider at any health care visit before pregnancy, at any prenatal visit, and at a postpartum checkup, respectively.

**What are the implications for public health practice?**

Routine assessment of smoking behaviors among pregnant and postpartum women can guide the development and implementation of evidence-based tobacco control measures.

## Abstract

Cigarette smoking during pregnancy increases the risk for pregnancy complications and adverse infant outcomes such as preterm delivery, restricted fetal growth, and infant death. Health care provider counseling can support smoking cessation. Data from the 2021 Pregnancy Risk Assessment Monitoring System were analyzed to estimate the prevalence of smoking before, during, and after pregnancy; quitting smoking during pregnancy; and whether health care providers asked about cigarette smoking before, during, and after pregnancy among women with a recent live birth. In 2021, the prevalence of cigarette smoking was 12.1% before pregnancy, 5.4% during pregnancy, and 7.2% during the postpartum period; 56.1% of women who smoked before pregnancy quit smoking while pregnant. Jurisdiction-specific prevalences of smoking ranged from 3.5% to 20.2% before pregnancy, 0.4% to 11.0% during pregnancy, and 1.0% to 15.1% during the postpartum period. Among women with a health care visit during the associated period, the percentage of women who reported that a health care provider asked about smoking was 73.7% at any health care visit before pregnancy, 93.7% at any prenatal care visit, and 57.3% at a postpartum checkup. Routine assessment of smoking behaviors among pregnant and postpartum women can guide the development and implementation of evidence-based tobacco control measures at the jurisdiction and health care–system level to reduce smoking among pregnant and postpartum women.

## Introduction

Maternal smoking during pregnancy increases the risk for pregnancy complications, including placenta previa, placental abruption, and premature rupture of membranes, and adverse infant outcomes such as cleft lip and palate, infant death, stillbirth, preterm delivery, restricted fetal growth, and sudden infant death syndrome (SIDS) (*1*). Smoking before pregnancy can impair fertility, and smoking after pregnancy increases the risk for SIDS and childhood respiratory infections (*1*). Jurisdictions can implement evidence-based strategies to reduce smoking, including among women of reproductive age (*2*). The U.S. Preventive Services Task Force (USPSTF) recommends that health care providers ask all adults, including pregnant women, about tobacco use, advise them to quit, and provide support for tobacco cessation interventions (*3*). This report assesses jurisdiction-level prevalence of cigarette smoking before, during, and after pregnancy, and whether health care providers asked about cigarette use at health care visits before, during, and after pregnancy.

## Methods

### Data Source

The Pregnancy Risk Assessment Monitoring System (PRAMS) is a population-based, jurisdiction-specific surveillance system that collects information on self-reported behaviors and experiences before, during, and after pregnancy among women with a recent live birth.* Women are surveyed by U.S. mail or by telephone 2–6 months after delivery (*4*). Maternal age, race and ethnicity, and education were obtained from the birth certificate. Health insurance coverage and history of depression before pregnancy were derived from the PRAMS questionnaire.^†^

### Descriptive and Statistical Analyses

The analysis includes 36,493 women (1,854,527 weighted) from 37 jurisdictions^§^ with a ≥50% response rate during 2021. This report presents data on measures of the following smoking behaviors before, during, and after pregnancy: 1) smoking during the 3 months before pregnancy, 2) smoking during the last 3 months of pregnancy, 3) quitting smoking during the last 3 months of pregnancy among women who smoked during the 3 months before pregnancy, and 4) smoking during the postpartum period (assessed at the time of questionnaire completion).^¶^^,^** Respondents with health care visits during the associated period (any health care visit during the 12 months before pregnancy, any prenatal care visit, and a postpartum checkup) reported whether a health care provider asked about cigarette smoking.^††^^,^^§§^

Prevalence of smoking behaviors and whether a health care provider asked about cigarette use were estimated by jurisdiction and demographic characteristics. All analyses were conducted using *SAS* software (version 9.4; *SAS* Institute). PRAMS data are weighted at the jurisdiction level; prevalence estimates and 95% CIs were calculated, and nonoverlapping CIs were considered statistically significant.^¶¶^ This study was reviewed and approved by the Institutional Review Boards at CDC and each participating PRAMS site.***

## Results

### Characteristics of Respondents and Smoking Behaviors

During 2021, 12.1% of surveyed women with a recent live birth reported smoking cigarettes during the 3 months before pregnancy, 5.4% smoked during the last 3 months of pregnancy, and 7.2% smoked during the postpartum period (Table 1). Among women who smoked during the 3 months before pregnancy, 56.1% quit smoking during pregnancy. The prevalence of smoking before pregnancy ranged from 3.5% in Puerto Rico to 20.2% in West Virginia; during pregnancy, from 0.4% in Puerto Rico to 11.0% in Maine; and during the postpartum period, from 1.0% in Puerto Rico to 15.1% in West Virginia. The prevalence of quitting smoking during pregnancy ranged from 35.9% in Wyoming to 87.9% in Puerto Rico. The following groups of women reported higher prevalences of smoking during pregnancy: non-Hispanic American Indian or Alaska Native (AI/AN) women, those who were Medicaid-insured for prenatal care, those who had completed ≤12 years of education, and those with a history of depression before pregnancy (Table 2).

**TABLE 1:** Prevalence of smoking before, during, and after pregnancy, and quitting smoking during pregnancy among women with a recent live birth, by jurisdiction — Pregnancy Risk Assessment Monitoring System, 2021*

Jurisdiction	No. of respondents	Weighted % (95% CI)			
		Smoked before pregnancy^†^	Smoked during pregnancy^§^	Quit smoking during pregnancy^¶^	Smoked during postpartum period**
**All jurisdictions**	**36,493**	**12.1 (11.6–12.7)**	**5.4 (5.0–5.8)**	**56.1 (53.7–58.5)**	**7.2 (6.8–7.6)**
Alabama	697	15.0 (12.1–17.8)	5.4 (3.5–7.2)	63.7 (53.3–74.2)	8.2 (5.9–10.5)
Arkansas	842	19.3 (15.3–23.3)	10.1 (7.1–13.2)	48.9 (37.3–60.6)	12.1 (8.9–15.4)
Colorado	1,261	9.8 (7.9–11.7)	3.5 (2.3–4.7)	66.5 (56.7–76.2)	4.4 (3.1–5.7)
Connecticut	1,328	8.9 (7.0–10.9)	2.9 (1.8–4.1)	68.8 (58.0–79.5)	5.2 (3.7–6.7)
Delaware	834	14.7 (11.9–17.4)	8.2 (6.1–10.3)	44.0 (33.7–54.2)	10.3 (8.0–12.7)
District of Columbia	500	8.4 (5.5–11.4)	3.4 (1.4–5.5)	59.4 (40.6–78.2)	5.0 (2.6–7.4)
Georgia	785	10.1 (7.3–12.9)	4.6 (2.7–6.6)	57.0 (42.3–71.7)	6.2 (4.0–8.5)
Hawaii	1,349	9.4 (7.3–11.4)	3.7 (2.3–5.0)	61.1 (49.7–72.5)	3.8 (2.5–5.1)
Illinois	1,119	12.3 (10.1–14.5)	4.3 (3.0–5.6)	65.2 (56.2–74.2)	6.7 (5.1–8.4)
Kansas	1,136	15.3 (12.6–18.0)	8.1 (6.1–10.2)	46.6 (36.9–56.2)	8.7 (6.7–10.8)
Louisiana	670	13.5 (10.7–16.4)	6.1 (4.0–8.2)	55.0 (43.3–66.7)	8.3 (6.0–10.7)
Maine	790	19.8 (16.2– 23.3)	11.0 (8.2–13.9)	44.2 (34.0–54.4)	13.3 (10.2–16.3)
Massachusetts	1,321	8.2 (6.0–10.5)	3.7 (2.1–5.3)	55.2 (40.7–69.7)	4.7 (2.9–6.5)
Michigan	1,374	16.4 (13.8–18.9)	8.5 (6.5–10.4)	48.2 (39.5–56.8)	10.0 (8.0–12.1)
Minnesota	634	12.4 (8.5–16.2)	4.5 (2.1–6.8)	64.2 (48.2–80.3)	6.7 (3.7–9.8)
Mississippi	886	15.7 (12.7–18.7)	8.3 (6.0–10.5)	46.8 (36.4–57.2)	11.1 (8.5–13.6)
Missouri	832	16.4 (13.5– 19.4)	8.7 (6.6–10.9)	47.9 (38.0–57.7)	10.8 (8.4–13.2)
Montana	1,169	19.4 (17.0–21.8)	9.5 (7.7–11.3)	51.0 (43.9–58.1)	10.8 (8.9–12.7)
Nebraska	1,226	11.8 (9.3–14.3)	4.4 (2.9–6.0)	62.3 (51.4–73.3)	7.4 (5.4–9.3)
New Jersey	942	7.0 (5.3–8.7)	2.2 (1.3–3.1)	70.6 (59.4–81.8)	3.9 (2.7–5.1)
New Mexico	1,064	11.8 (9.8–13.8)	5.0 (3.7–6.4)	59.2 (50.3–68.2)	6.5 (4.9–8.0)
New York^††^	868	13.4 (10.3–16.5)	5.6 (3.3–7.8)	57.8 (45.0–70.5)	7.4 (4.9–9.8)
New York City	1,263	4.5 (3.2–5.8)	0.6 (0.2–1.0)	87.4 (78.3–96.6)	2.2 (1.3–3.1)
North Dakota	586	15.9 (12.4–19.4)	6.6 (4.3–9.0)	58.3 (46.3–70.4)	9.0 (6.2–11.8)
Oklahoma	1,460	15.5 (12.6–18.4)	5.9 (4.0–7.8)	61.7 (51.6–71.8)	9.7 (7.2–12.1)
Oregon	1,878	10.9 (8.5–13.2)	4.2 (2.7–5.7)	61.4 (50.0–72.7)	6.5 (4.6–8.4)
Pennsylvania	934	15.8 (12.8–18.8)	8.7 (6.4–11.0)	44.8 (34.4–55.1)	10.0 (7.6–12.4)
Puerto Rico	965	3.5 (2.2–4.8)	0.4 (0–0.9)	87.9 (74.9–100.0)	1.0 (0.3–1.6)
South Dakota	1,026	19.5 (17.0–22.1)	9.8 (7.8–11.8)	49.4 (42.1–56.7)	13.4 (11.2–15.5)
Tennessee	633	16.1 (12.8–19.4)	7.1 (4.8–9.4)	55.6 (44.5–66.8)	9.5 (6.9–12.1)
Utah	1,259	6.1 (4.7–7.5)	2.3 (1.4–3.2)	62.2 (50.3–74.1)	2.9 (2.0–3.9)
Vermont	960	16.8 (14.3–19.3)	8.1 (6.3–10.0)	52.6 (44.4–60.9)	10.1 (8.0–12.2)
Virginia	939	10.6 (7.1–14.1)	4.8 (2.3–7.2)	63.5 (46.6–80.5)	6.0 (3.2–8.7)
Washington	1,147	8.3 (6.2–10.4)	4.2 (2.7–5.8)	52.1 (38.7–65.5)	5.0 (3.3–6.7)
West Virginia	604	20.2 (16.4–24.1)	10.3 (7.5–13.2)	49.0 (38.5–59.5)	15.1 (11.8–18.5)
Wisconsin	764	10.3 (7.4–13.1)	5.8 (3.6–8.1)	42.7 (27.8–57.6)	7.6 (5.0–10.2)
Wyoming	448	16.3 (12.2–20.5)	10.5 (6.9–14.1)	35.9 (22.6–49.1)	12.5 (8.6–16.3)

**Abbreviation:** PRAMS = Pregnancy Risk Assessment Monitoring System.

* All jurisdictions met the minimum overall response rate threshold of ≥50%.

^†^ Defined as any smoking during the 3 months before pregnancy.

^§^ Defined as any smoking during the last 3 months of pregnancy.

^¶^ Defined as no smoking during the last 3 months of pregnancy among women who smoked during the 3 months before pregnancy.

** Defined as any smoking at the time of PRAMS questionnaire administration (approximately 2–6 months after delivery).

^††^ New York data do not include New York City.

**TABLE 2:** Prevalence of smoking before, during, and after pregnancy, and quitting smoking during pregnancy among women with a recent live birth, by maternal characteristics — Pregnancy Risk Assessment Monitoring System, 2021*

Characteristic	No. of respondents	Weighted % (95% CI)			
		Smoked before pregnancy^†^	Smoked during pregnancy^§^	Quit smoking during pregnancy^¶^	Smoked during postpartum period**
**All women with a recent live birth**	**36,493**	**12.1 (11.6−12.7)**	**5.4 (5.0–5.8)**	**56.1 (53.7–58.5)**	**7.2 (6.8–7.6)**
**Age group, yrs**					
<20	1,512	12.6 (9.8–15.4)	4.3 (2.6–5.9)	66.2 (55.1–77.3)	7.2 (5.0–9.4)
20–24	6,100	15.8 (14.2–17.4)	5.8 (4.9–6.8)	63.0 (57.7–68.2)	8.6 (7.4–9.8)
25–34	21,263	11.9 (11.2–12.6)	5.6 (5.1–6.1)	53.9 (50.8–57.1)	7.2 (6.6–7.8)
≥35	7,617	9.7 (8.6–10.8)	4.8 (4.0–5.6)	52.1 (46.2–58.0)	6.1 (5.2–6.9)
**Race and ethnicity^††^**					
American Indian or Alaska Native	1,361	31.4 (26.3–36.5)	16.6 (11.3–21.9)	47.4 (37.3–57.5)	21.8 (16.6–27.0)
Asian or Pacific Islander	2,798	2.9 (1.9–3.8)	0.5 (0.2–0.9)	80.7 (69.9–91.5)	1.2 (0.5–1.8)
Black or African American	5,703	9.4 (8.2–10.5)	3.9 (3.2–4.5)	60.2 (54.2–66.2)	6.9 (5.9–8.0)
White	16,695	14.9 (14.1–15.7)	7.1 (6.5–7.7)	53.5 (50.4–56.5)	8.9 (8.2–9.5)
Hispanic or Latino	7,431	6.7 (5.8–7.5)	2.0 (1.5–2.4)	71.4 (65.4–77.3)	2.9 (2.4–3.5)
Another race or multiple races	2,206	16.4 (13.4–19.4)	8.7 (6.1–11.3)	47.2 (37.6–56.8)	9.8 (7.2–12.3)
**Education, yrs**					
<12	4,000	19.6 (17.5–21.6)	11.8 (10.1–13.5)	40.3 (34.7–45.9)	13.4 (11.7–15.2)
12	8,678	19.9 (18.5–21.3)	9.5 (8.5–10.5)	52.3 (48.4–56.3)	12.9 (11.7–14.1)
>12	23,561	8.0 (7.5–8.6)	2.9 (2.5–3.2)	65.8 (62.4–69.2)	4.1 (3.7–4.5)
**Health insurance coverage^§§^**					
Private	20,025	7.1 (6.5–7.7)	2.2 (1.9–2.5)	69.9 (66.0–73.8)	3.3 (2.9–3.7)
Medicaid	13,038	21.5 (20.3–22.7)	11.4 (10.4–12.3)	48.0 (44.9–51.2)	14.5 (13.4–15.5)
Other insurance**^¶¶^**	751	8.1 (4.6–11.6)	1.9 (0.7–3.1)	77.3 (62.1–92.4)	4.3 (1.6–7.0)
Uninsured	263	10.5 (4.5–16.5)	6.5 (0.9–12.1)	38.2 (12.3–64.1)	5.9 (0.7–11.1)
History of depression before pregnancy***					
Yes	6,358	27.1 (25.3–29.0)	14.6 (13.0–16.1)	48.1 (44.0–52.2)	18.1 (16.5–19.8)
No	29,775	9.2 (8.7–9.7)	3.6 (3.3–4.0)	60.9 (57.9–63.8)	5.1 (4.7–5.5)

**Abbreviation:** PRAMS = Pregnancy Risk Assessment Monitoring System.

* Data were aggregated for the following 37 PRAMS jurisdictions with a response rate of ≥50% during 2021: Alabama, Arkansas, Colorado, Connecticut, Delaware, District of Columbia, Georgia, Hawaii, Illinois, Kansas, Louisiana, Maine, Massachusetts, Michigan, Minnesota, Mississippi, Missouri, Montana, Nebraska, New Jersey, New Mexico, New York, New York City, North Dakota, Oklahoma, Oregon, Pennsylvania, Puerto Rico, South Dakota, Tennessee, Utah, Vermont, Virginia, Washington, West Virginia, Wisconsin, and Wyoming.

^†^ Defined as any smoking during the 3 months before pregnancy.

^§^ Defined as any smoking during the last 3 months of pregnancy.

^¶^ Defined as no smoking during the last 3 months of pregnancy among women who smoked during the 3 months before pregnancy.

** Defined as any smoking at the time of PRAMS questionnaire administration (approximately 2–6 months after delivery).

^††^ Persons of Hispanic or Latino (Hispanic) origin might be of any race but are categorized as Hispanic; all racial groups are single-race non-Hispanic unless otherwise specified. Another race or multiple races include those with more than one race or other race.

^§§^ Determined from women’s reported coverage during prenatal care.

^¶¶^ Other health insurance coverage includes Tricare, other military health insurance, Indian Health Service, or state-specific State Children’s Health Insurance Program or Children’s Health Insurance Program.

*** History of depression before pregnancy was defined as depression during the 3 months before pregnancy as reported in PRAMS.

### Health Care Provider Asking About Smoking

Among women with a health care visit during the associated period, 73.7% reported that a health care provider asked about current cigarette smoking at a health care visit during the 12 months before pregnancy, 93.7% reported that a health care provider asked about cigarette smoking at any prenatal care visit, and 57.3% reported that a health care provider asked about cigarette smoking at a postpartum checkup (Table 3). The percentage of women who were asked about cigarette smoking by a health care provider at a postpartum checkup was lower in the following groups: women aged ≥35 years, those who had completed >12 years of education, those without a history of depression, and those who did not smoke before pregnancy.

**TABLE 3:** Prevalence of a health care provider asking about current cigarette smoking before, during, and after pregnancy among women with a recent live birth, by selected maternal characteristics (N = 36,493) — Pregnancy Risk Assessment Monitoring System, 2021*^,^**^†^**

Characteristic	Asked about cigarette smoking, weighted % (95% CI)		
	At any visit 12 months before pregnancy^§ ^n = 23,539	At any prenatal care visit^¶ ^n = 35,513	During a postpartum checkup** n = 31,866
**All women with a recent live birth**	**73.7 (72.8–74.6)**	**93.7 (93.3–94.1)**	**57.3 (56.4–58.1)**
**Age group, yrs**			
<20	78.9 (73.8–83.9)	94.5 (92.6–96.4)	73.8 (69.7–78.0)
20–24	76.5 (74.1–78.9)	92.9 (91.8–94.1)	66.7 (64.6–68.9)
25–34	73.9 (72.8–75.0)	94.3 (93.8–94.7)	56.1 (55.0–57.2)
≥35	71.1 (69.2–73.0)	92.8 (91.8–93.7)	50.5 (48.7–52.4)
**Race and ethnicity^††^**			
American Indian or Alaska Native	76.0 (66.7–85.2)	97.3 (96.2–98.4)	76.2 (72.1–80.2)
Asian or Pacific Islander	62.3 (58.4–66.3)	91.7 (89.9–93.5)	54.7 (51.4–58.1)
Black or African American	76.0 (73.5–78.5)	92.9 (91.7–94.0)	67.8 (65.6–70.1)
White	73.3 (72.1–74.4)	94.2 (93.7–94.7)	51.6 (50.5–52.8)
Hispanic or Latino	77.0 (74.8–79.2)	93.1 (92.2–94.1)	68.7 (66.8–70.5)
Another race or multiple races	76.9 (72.8–81.1)	95.5 (93.7–97.3)	57.6 (53.0–62.3)
**Education, yrs**			
<12	74.2 (70.2–78.1)	89.5 (87.8–91.2)	72.8 (70.0–75.6)
12	76.1 (74.1–78.2)	93.4 (92.5–94.3)	67.4 (65.6–69.2)
>12	73.0 (72.0–74.0)	94.5 (94.1–95.0)	51.7 (50.7–52.7)
**Health insurance coverage^§§^**			
Private	73.3 (72.3–74.3)	94.4 (93.9–94.9)	50.6 (49.5–51.7)
Medicaid	76.5 (74.8–78.3)	94.6 (93.9–95.2)	69.1 (67.7–70.6)
Other insurance^¶¶^	58.7 (48.5–68.9)	79.4 (74.4–84.5)	53.8 (46.7–60.9)
Uninsured	73.7 (59.6–87.7)	93.0 (88.8–97.1)	73.7 (64.6–82.8)
History of depression before pregnancy***			
Yes	81.4 (79.6–83.2)	94.9 (94.0–95.8)	61.2 (59.0–63.3)
No	72.1 (71.1–73.1)	93.6 (93.1–94.0)	56.5 (55.5–57.4)
**Smoked before pregnancy^†††^**			
Yes	85.6 (83.3–87.9)	97.3 (96.6–98.0)	69.7 (67.3–72.2)
No	72.2 (71.3–73.2)	93.3 (92.8–93.7)	55.8 (54.9–56.7)

**Abbreviation:** PRAMS = Pregnancy Risk Assessment Monitoring System.

* Data were aggregated for the following 37 PRAMS jurisdictions with a response rate of ≥50% during 2021: Alabama, Arkansas, Colorado, Connecticut, Delaware, District of Columbia, Georgia, Hawaii, Illinois, Kansas, Louisiana, Maine, Massachusetts, Michigan, Minnesota, Mississippi, Missouri, Montana, Nebraska, New Jersey, New Mexico, New York, New York City, North Dakota, Oklahoma, Oregon, Pennsylvania, Puerto Rico, South Dakota, Tennessee, Utah, Vermont, Virginia, Washington, West Virginia, Wisconsin, and Wyoming.

^†^ Although 99% of women with a recent live birth attended prenatal care visits, 33% did not have a health care visit during the 12 months before pregnancy and 9% did not have a postpartum care visit.

^§^ Among women who reported that they had a health care visit with a doctor, nurse, or other health care worker, including a dental or mental health worker during the 12 months before pregnancy and provided a response to the PRAMS question about a health care provider asking about cigarette use.

^¶^ Among women who reported a prenatal care visit and provided a response to the PRAMS question about a health care provider asking about cigarette use.

** Among women who reported having had a postpartum checkup and provided a response to the PRAMS question about a health care provider asking about cigarette use.

^††^ Persons of Hispanic or Latino (Hispanic) origin might be of any race but are categorized as Hispanic; all racial groups are single-race non-Hispanic unless otherwise specified. Another race or multiple races include those with more than one race or other race.

^§§^ Determined from women’s reported coverage during prenatal care.

^¶¶^ Other health insurance coverage includes Tricare, other military health insurance, Indian Health Service, or state-specific State Children’s Health Insurance Program or Children’s Health Insurance Program.

*** History of depression before pregnancy was defined as depression during the 3 months before pregnancy as reported in PRAMS.

^†††^ Smoking before pregnancy was defined as any smoking during the 3 months before pregnancy as reported in PRAMS.

## Discussion

This analysis found that during 2021, one in 18 women with a recent live birth smoked during pregnancy, with wide variation by jurisdiction (range = 0.4%–11.0%). Although 56.1% of women who smoked before pregnancy quit during pregnancy, approximately one in 13 smoked during the postpartum period. USPSTF recommends that health care providers ask all adult patients about tobacco use, including pregnant and postpartum women (*3*). However, although 93.7% of women reported being asked about cigarette smoking during a prenatal care visit, only 57.3% reported being asked about cigarette smoking at a postpartum checkup. In addition, only 69.7% of women who reported smoking before pregnancy were asked about cigarette smoking during the postpartum period. Assessment of tobacco use by health care providers is an important first step in improving quitting success, affording an opportunity to follow up with patients about their readiness to quit and to provide access to cessation resources (*3*). Guidance for the comprehensive postpartum visit includes screening for tobacco use, with counseling regarding relapse during the postpartum period among women who quit smoking during pregnancy (*5*).

Both behavioral and pharmacological interventions are effective methods to increase smoking cessation (*3*). For nonpregnant adults, smoking cessation medications approved by the Food and Drug Administration can improve the likelihood of successfully quitting smoking and result in higher rates of quitting when used in combination with behavioral cessation counseling; however, these medications are not recommended during pregnancy because of insufficient evidence that nicotine replacement therapy does not affect birth outcomes (*3*). Insurance coverage for comprehensive and barrier-free smoking cessation counseling and treatments is cost-effective.^†††^ Beginning in 2010, Medicaid programs were required to cover tobacco cessation services for pregnant women without cost sharing (*6*). Health care providers can also refer persons who smoke to toll-free national Quitline telephone numbers^§§§^ to link patients to telephone-based cessation resources. In addition to health care–related strategies, effective tobacco control measures at the population level, such as tobacco taxes, public health campaigns, and smoke-free policies, support smoking cessation among adults (*2*). Studies have demonstrated the benefits of strategies such as public health campaigns (*7*) and Quitlines (*8*) among pregnant women.

The prevalence of smoking during the perinatal period has decreased. Analyses using PRAMS data have demonstrated a decreased prevalence of smoking before, during, and after pregnancy, as well as an increase in quitting during pregnancy, from 2000 to 2020 (*9*). Estimates of smoking during pregnancy from PRAMS differ from other data sources; however, methods also differ. According to the 2020 National Survey on Drug Use and Health, 8.4% of pregnant women used tobacco products.^¶¶¶^ Based on 2021 birth certificate data, 4.6% of women who gave birth in the United States smoked during pregnancy.**** Similar to the current report, the National Center for Health Statistics report found the prevalence of smoking during pregnancy was higher among younger age groups and AI/AN women, with variation by jurisdiction. New York City and Puerto Rico were the only PRAMS jurisdictions that met the Healthy People 2020 goal of reducing prenatal smoking to 1.4%.^††††^

Comprehensive tobacco control measures at the state and jurisdiction level have been demonstrated to reduce smoking at the population level (*2*). For example, in jurisdictions with low levels of prenatal smoking (New York City and Puerto Rico), cigarette excise taxes were above $4 per pack and comprehensive smoke-free indoor air legislation had been enacted jurisdiction-wide.^§§§§^ In contrast, among PRAMS jurisdictions with the highest levels of prenatal smoking (Maine, West Virginia, and Wyoming), cigarette excise taxes were ≤$2 per pack. West Virginia and Wyoming had no statewide comprehensive smoke-free indoor air legislation.

### Limitations

The findings in this report are subject to at least six limitations. First, women might underreport socially undesirable behaviors such as smoking during pregnancy or overreport socially desirable behaviors such as quitting smoking during pregnancy. Second, because PRAMS responses are obtained 2–6 months postpartum, they might be affected by recall bias. Third, smoking prevalences in this report did not include other types of tobacco use, such as electronic vapor products, which likely results in an underestimate of the prevalence of tobacco use (*10*). Fourth, the reported prevalence of smoking during pregnancy was limited to the timeframe of the last 3 months of pregnancy and did not capture smoking during other periods in pregnancy. Fifth, only women who attended a health care visit could be queried by their provider regarding their smoking status. Finally, the generalizability of the findings of this report is limited to PRAMS jurisdictions included in this analysis.

### Implications for Public Health Practice

Routine assessment of smoking behaviors among pregnant and postpartum women can guide the development and implementation of evidence-based tobacco control measures at the jurisdiction and health care–system level to reduce smoking.^¶¶¶¶^ Health care providers can increase their efforts to assess smoking status among all adults, including pregnant and postpartum women, provide cessation counseling and medication when appropriate, refer women for more intensive cessation counseling, and promote available cessation services. Jurisdictions can support evidence-based tobacco control measures to reduce smoking among pregnant and postpartum women.

## Footnotes

* Not all pregnant persons identify as women. “Women” is used in this report because PRAMS data are sampled from birth certificates of women with a recent live birth.

† Health insurance coverage was defined from women’s reported coverage during prenatal care.

§ Alabama, Arkansas, Colorado, Connecticut, Delaware, District of Columbia, Georgia, Hawaii, Illinois, Kansas, Louisiana, Maine, Massachusetts, Michigan, Minnesota, Mississippi, Missouri, Montana, Nebraska, New Jersey, New Mexico, New York, New York City, North Dakota, Oklahoma, Oregon, Pennsylvania, Puerto Rico, South Dakota, Tennessee, Utah, Vermont, Virginia, Washington, West Virginia, Wisconsin, and Wyoming.

¶ PRAMS questions used to calculate cigarette smoking behavior measures included 1) “In the 3 months before you got pregnant, how many cigarettes did you smoke on an average day?”; 2) “In the last 3 months of your pregnancy, how many cigarettes did you smoke on an average day?”; and 3) “How many cigarettes do you smoke on an average day now?” Based on responses to these questions (e.g., “at least one cigarette per day on an average day”), dichotomous variables were created to define any cigarette smoking before pregnancy, during pregnancy, and during the postpartum period. Data on smoking during other time points in pregnancy are not collected.

**
https://www.cdc.gov/prams/questionnaire.htm

†† PRAMS questions used to calculate health care providers asking about cigarette smoking included 1) “During any of your health care visits in the 12 months before you got pregnant, did a doctor, nurse, or other health care worker ask you if you were smoking cigarettes?”; 2) “During any of your prenatal care visits, did a doctor, nurse, or other health care worker ask you if you were smoking cigarettes?”; and 3) “During your postpartum checkup, did a doctor, nurse, or other health care worker ask you if you were smoking cigarettes?” Women could have had more than one health care visit during the postpartum period.

§§ Among women with a recent live birth, 33% did not have a health care visit during the 12 months before pregnancy, 1% did not attend prenatal care visits, and 9% did not have a postpartum care visit. Percentages are reported among those who attended a visit during the relevant period and provided a response to the question about a health care provider asking about cigarette use.

¶¶ Each participating jurisdiction selects a monthly stratified sample of women from birth certificate records. Data were weighted to adjust for noncoverage and nonresponse and to represent the total population of women with a live birth in each jurisdiction in 2021. PRAMS aggregate data are not weighted to provide national estimates. The analyses were conducted using survey analysis procedures to account for the complex sampling design of PRAMS.

*** 45 C.F.R. part 46, 21 C.F.R. part 56.

††† Barrier-free refers to health insurance coverage that removes or reduces barriers to accessing cessation treatments (e.g., copayments, coinsurance, deductibles, and prior authorization). https://archive.cdc.gov/#/details?url=https://www.cdc.gov/tobacco/quit_smoking/cessation/coverage/index.htm

§§§
https://www.cdc.gov/tobacco/campaign/tips/quit-smoking/index.html

¶¶¶
https://www.samhsa.gov/data/sites/default/files/reports/slides-2020-nsduh/2020NSDUHWomenSlides072522.pdf

****
https://www.cdc.gov/nchs/data/databriefs/db458.pdf

††††
https://wayback.archive-it.org/5774/20220415223525/https://www.healthypeople.gov/2020/topics-objectives/objective/mich-113

§§§§
https://www.cdc.gov/statesystem/statehighlights.html

¶¶¶¶
https://www.hhs.gov/sites/default/files/hhs-framework-support-accelerate-smoking-cessation-2024.pdf
